# Managing antiretrovirals in co-infected patients in 2012

**DOI:** 10.1186/1742-4690-9-S1-I1

**Published:** 2012-05-25

**Authors:** Jürgen Rockstroh

**Affiliations:** 1 University of Bonn, Germany

## 

In patients with HIV and hepatitis B or C co-infection initiation of early HAART therapy is clearly recommended by the international guidelines reflecting the overall survival benefit with regard not only to HIV but also clinical endpoints of liver disease in the co-infected patient population. In patients with hepatitis C co-infection administration of successful HAART is associated with less inflammation in liver biopsies and hence, over time less fibrosis generation. Obviously, drugs which are dually active against HIV and HBV do not only prevent HIV disease progression, but also decrease the progression of chronic hepatitis B related liver disease. Nevertheless, recent reports on the emergence of non cirrhotic portal hypertension as a rare cause of upper gastrointestinal bleeding in HIV patients most likely following prolonged didanosin exposure have raised questions with regard to potential drug toxicity as sequelae of HAART causing possible damage to the liver and the portal vascular system. Under consideration of metabolic changes including dislipidemia and insulin resistance caused by various components of different drug classes the question also remains in how far risk for fatty liver disease may rise after decades of HAART administration. Therefore, close surveillance programs and monitoring are required to answer the long term safety questions around HAART administration and liver safety. Nevertheless, the clear demonstration of overall increased survival under HAART in patients with concomitant hepatitis and HIV clearly underlines that the benefits of HAART outweighs potential toxicity risks. Also with the knowledge obtained around mitochondrial toxicity observed under D-nucleoside therapy treatment algorithms for co-infected patients now specifically refrain from using these drugs in particular. With the advent of new drug classes with low hepatotoxicity profiles new combinations arise which potentially improved liver safety profile over time. But again, long term data will be needed to eventually decide what are the best HIV treatment options in patients with concomitant liver disease.

